# Detection of Deltamethrin resistance level and association between sodium channel domain III gene mutation sites and drug resistance of *Rhipicephalus microplus*

**DOI:** 10.1515/biol-2025-1287

**Published:** 2026-05-13

**Authors:** Zhong-Bo Li, Liangxin Duan, Xin Liu, Tian Yang, Hui Hu, Min Xiang, Cui-qin Huang

**Affiliations:** College of Animal Science and Technology, HuaiHua Vocational and Technical College, Huaihua, Hunan Province 418000, China; Key Laboratory of Resistance Detection and Symbiotic Bacteria Utilization of Vector Organisms in Wuling Mountain Area, Huaihua 418000, China; Engineering Research Center for the Prevention and Control of Animal Original Zoonosis, College of Life Science, Fujian Province University, Longyan University, Longyan, Fujian Province 364012, China

**Keywords:** *Rhipicephalus microplus*, Deltamethrin, sodium channel domain III gene, adult immersion test, mutation site, resistance

## Abstract

To investigate the level of Deltamethrin resistance and mutation sites in the sodium iron channel gene in *Rhipicephalus microplus* (*R. microplus*) in Huaihua City, Hunan Province, and to examine the correlation between Deltamethrin resistance and mutation sites in the sodium iron channel gene of *R. microplus*. A total of 600 *R. microplus* were sampled from multiple yellow cattle farms in Huaihua City, Hunan Province from June to September 2022, and the level of resistance to Deltamethrin was determined in ticks using the adult immersion test. The sodium iron channel domain III gene was amplified in Deltamethrin-resistant and wild-type *R. microplus* using PCR assay. Following sequencing and sequence alignment, mutation sites were detected in bases. The sodium iron channel domain III gene in *R. microplus* was translated, and the signal peptide, transmembrane domain, and phosphorylation and glycosylation sites were detected in amino acid sequences. The tertiary structures of the sodium iron channel domain III protein of Deltamethrin-resistant and wild-type *R. microplus* were deduced and compared, and the association between mutation sites in bases and resistance to Deltamethrin was examined in *R. microplus* according the level of Deltamethrin resistance, sequence alignment and protein tertiary structure. The median (LC50) and 95 % lethal concentrations (LC95) of Deltamethrin were 121.39 mg/L and 952.61 mg/L against *R. microplus*, with a resistance factor of 9.24 and level II resistance. The sequence of the sodium ion channel domain III gene was 1,010 bp in size, and mutation sites were detected in two neighboring bases in the sequence of the sodium ion channel domain III gene in Deltamethrin-resistant *R. microplus*. Although no signal peptides were found in the sodium iron channel domain III protein of Deltamethrin-resistant or wild-type *R. microplus*, 6 trans-membrane domains, 42 phosphorylation sites and 8 glycosylation sites were identified, with a significant difference in the tertiary structure of the sodium iron.

## 1 Introduction

*Rhipicephalus microplus* (*R. microplus*) as an important and common hematophagous ectoparasite, is wide distribution in tropical and subtropical regions [1], [2], [3]. This parasite falls under the Ixodidae family and is classified within the *Rhipicephalus* genus [4], making it a key member of the second-largest group of disease vectors [5]. It has the capability to transmit extensive pathogens, including viruses, bacteria, fungi, and protozoa, to its hosts [6]. According to the Food and Agriculture Organization (FAO), approximately 85 % of global cattle populations are at risk of infestation by *R. microplus* [7]. Despite this, chemical control remains the predominant method for managing harmful organisms in agriculture and livestock farming [8], 9]. Among these chemicals, Deltamethrin – a type II pyrethroid insecticide – is widely favored ascribe to its high efficacy with minimal toxicity [10]. However, prolonged and improper use has led to significant environmental contamination and widespread resistance to this insecticide [11], 12]. Internationally, research and surveillance on Deltamethrin resistance in *R. microplus* have been conducted [13], 14], but domestic studies in this area remain limited. Therefore, there is an urgent need to investigate this issue further.

Presently, it is widely acknowledged that vector insects develop resistance to insecticides through three primary mechanisms: physiological, behavioral, and physiological-biochemical resistance [15]. Notably, physiological-biochemical resistance includes target-site resistance [16], which is characterized by genetic mutations in the voltage-gated sodium ion channels of insect nerve cell membranes. These mutations reduce sensitivity to pyrethroid and DDT insecticides, leading to resistance [17]. Studies have shown a direct link between mutations in sodium ion channel genes and resistance to these insecticides [18]. For instance, Martinez-Torres et al. identified the S6 segment of the sodium channel gene was found to harbor the L1014F mutation as the cause of resistance to pyrethroids and DDT in *Anopheles gambiae* and *Culex quinquefasciatus* [19]; Vudriko et al. found that mutations in domain II of the sodium ion channel gene in *Rhipicephalus decoloratus* directly contributed to resistance against synthetic pyrethroids [20]. Similarly, He et al. observed that mutations in domain III of the sodium ion channel gene in *R. microplus* influenced its resistance to Deltamethrin [21]. Additionally, other researches has demonstrated high homology in domain III sequences of sodium ion channels across various insect species [22].

Given the growing concern over insecticide resistance in *R. microplus*, a highly harmful arthropod, identifying mutations in the sodium ion channel gene is crucial for controlling its spread in China. This study focuses on *R. microplus* samples collected from the Huaihua region. Using the adult immersion method, we assessed the resistance of this population to Deltamethrin, determined the resistance level, and PCR-amplified and sequenced domain III of the sodium ion channel gene to detect mutation rates. The findings aim to provide valuable information and technical guidance for selecting appropriate insecticides against *R. microplus* in this region.

## 2 Materials and methods

### 2.1 Sample collection

A total of 942 adult female ticks, comprising 780 fully engorged and 162 partially engorged specimens, were gathered from yellow cattle in several livestock farms across the Huaihua area. These ticks were identified as *R. microplus*.

### 2.2 Key reagents

The genomic DNA extraction kit for animal tissues was obtained from Tiangen Biotech (Beijing) Co., Ltd. High-fidelity Taq polymerase was supplied by Takara Bioengineering (Dalian) Co., Ltd. Proteinase K and the 2000 DNA Marker were provided by Beijing TransGen Biotech Co., Ltd. Analytical grade acetone solution and 98 % Deltamethrin technical grade were both procured from Sinopharm Chemical Reagent Co., Ltd.

### 2.3 Adult immersion test

A total of 600 fully engorged adult female *R. microplus* ticks were randomly divided into six groups (labeled Group I to Group VI), with each group comprising 100 ticks. Group I functioned as the control group, whereas Groups II to VI were designated as experimental groups. Following the protocol established by Sharma et al. [23], we prepared a stock solution using acetone and subsequently diluted Deltamethrin to generate six concentration levels: 0 mg/L, 62.5 mg/L, 125 mg/L, 250 mg/L, 500 mg/L, and 1,000 mg/L. The control group was only exposed to the acetone solvent.

Each tick group underwent its respective treatment and was then transferred to an environmental chamber set at 80 ± 5 % relative humidity and 28 ± 1 °C temperature. These conditions were maintained for continuous cultivation over a period of 14 days. At the conclusion of this incubation period, tick viability was evaluated by gently stimulating them with a small brush. Ticks that failed to show any movement in response to this stimulation were categorized as non-viable.

### 2.4 Data processing and analysis

The mortality rates, including the corrected mortality rate, were calculated based on the survival status of the ticks. The resistance ratio (RR) was determined, whereas the lethal concentrations (LC50 and LC95) and their respective 95 % confidence intervals (CI) were determined by applying regression equation analysis to the probit transformed data of mortality. The dose response data was analysed by probit method using Graph Pad Prism 4 software. The resistance factors (RF) for field tick isolates were calculated as the quotient between LC50 of field ticks and LC50 of a susceptible strain of *R. microplus*. On the basis of RF, the resistance levels (RL) of *R. microplus* were classified as susceptible (RF ≤ 1.4), level I resistance (1.5 ≤ RF ≤ 5.0), level II resistance (5.1 ≤ RF ≤ 25.0), level III resistance (25.1 ≤ RF ≤ 40.0) and level IV resistance (RF > 40) [24].

### 2.5 Primers design

Based on the sodium ion channel gene sequence of *R. microplus* (AF134216), a pair of primers was designed using Primer 5.0 software: Vgsc-F: 5′-GAA​GAT​GTG​GAC​ACA​GAC​AAG​CTG​G-3′, Vgsc-R: 5′-CCC​ATT​TTC-TTC​ATG​GCG​TTA​TAG-3′. These primer sequences were synthesized by Sangon Biotech (Shanghai) Co., Ltd.

### 2.6 DNA extraction and detection

After a 14-day rearing period, six ticks were randomly selected from each of the surviving groups (I to VI), one tick per group, labeled RM-1 to RM-6. These surviving specimens represented the survival phenotype under their respective acaricide concentrations. Genomic DNA was extracted from these samples following the instructions provided with the animal tissue genomic DNA extraction kit. Each DNA extract (2 μL) was then subjected to electrophoresis on a 1.5 % agarose gel containing ethidium bromide for verification. Positive DNA extracts were stored at −20 °C for future use.

### 2.7 Amplification, detection, and preservation of sodium ion channel domain III gene

DNA amplification was performed using extracted genomic DNA as template to amplify the domain III region of *R. microplus* sodium channel gene. PCR reactions were carried out in a 50 μL system containing 25 μL Taq DNA polymerase, 1 μL each of forward and reverse primers, 1 μL template DNA, and 22 μL double-distilled water. The thermal cycling conditions comprised an initial denaturation at 95 °C for 5 min, followed by 35 cycles of 95 °C for 30 s, 54 °C for 30 s, and 72 °C for 1 min, with a final extension at 72 °C for 5 min. PCR products were verified by electrophoresis on ethidium bromide-stained 1.5 % agarose gels, with 5 μL aliquots loaded per sample. Confirmed amplicons were preserved at −20 °C for future use.

### 2.8 Sequencing, BLAST comparison, and ORF reading frame sequence determination

Following PCR amplification, the target gene fragments were sent to Shanghai Sangon Biotech Company for sequencing in both forward and reverse directions (three biological replicates per sample) to obtain the sodium ion channel domain III gene sequence of *R. microplus*. The resulting sequences were inspected using Chromas software, and the original sequences were compared against NCBI databases using BLAST to confirm their identity as target gene sequences. The open reading frame (ORF) sequence of the obtained gene was determined using the EXPASY online tool.

### 2.9 Genetic evolutionary analysis

To analyze the genetic distribution of the sodium ion channel domain III gene across various arthropods, we downloaded relevant sequences from NCBI for *Rhipicephalus annulatus* (MH607137), *Dermacentor andersoni* (XM_055077119), *Dermacentor silvarum* (XM_049662144), *Ixodes scapularis* (XM_042289002), *Varroa jacobsoni* (XM_022830057), *Varroa destructor* (XM_022807031), *Neoseiulus californicus* (OP612720), *Amblyseius swirskii* (ON552241), and *Culex pipiens pallens* (AB453978). Using Clustal X and PhyML 3.0, we performed maximum likelihood (ML) analysis to construct an unrooted genetic evolutionary tree with FigTree v1.3.1.

### 2.10 Analysis of signal peptides, transmembrane domains, phosphorylation, and glycosylation sites

The signal peptides and transmembrane domains of the sodium channel domain III protein in various genotypes of *R. microplus* were predicted using SignalP 5.0 and TMHMM, respectively. Additionally, phosphorylation and glycosylation sites were predicted using NetPhos 3.1 and NETCGlyc 1.0.

### 2.11 Tertiary structure analysis of sodium channel domain III protein

The tertiary structure of the sodium channel domain III protein in both resistant and non-resistant *R. microplus* was predicted using PRABI and SWISS-MODEL. Coverage and similarity were calculated and compared to identify structural differences.

### 2.12 Detection of base mutation sites and correlation with resistance

The ORF sequences of all sodium ion channel domain III genes were aligned using Clustal X to detect base mutation sites in the resistant tick samples. Combining these results with tick resistance data, the tertiary structure of the sodium channel domain III protein in resistant *R. microplus*, and relevant literature, we investigated the correlation between detected mutations and resistance to Deltamethrin.

## 3 Results

### 3.1 Adult immersion test outcomes

**Figure 1:**
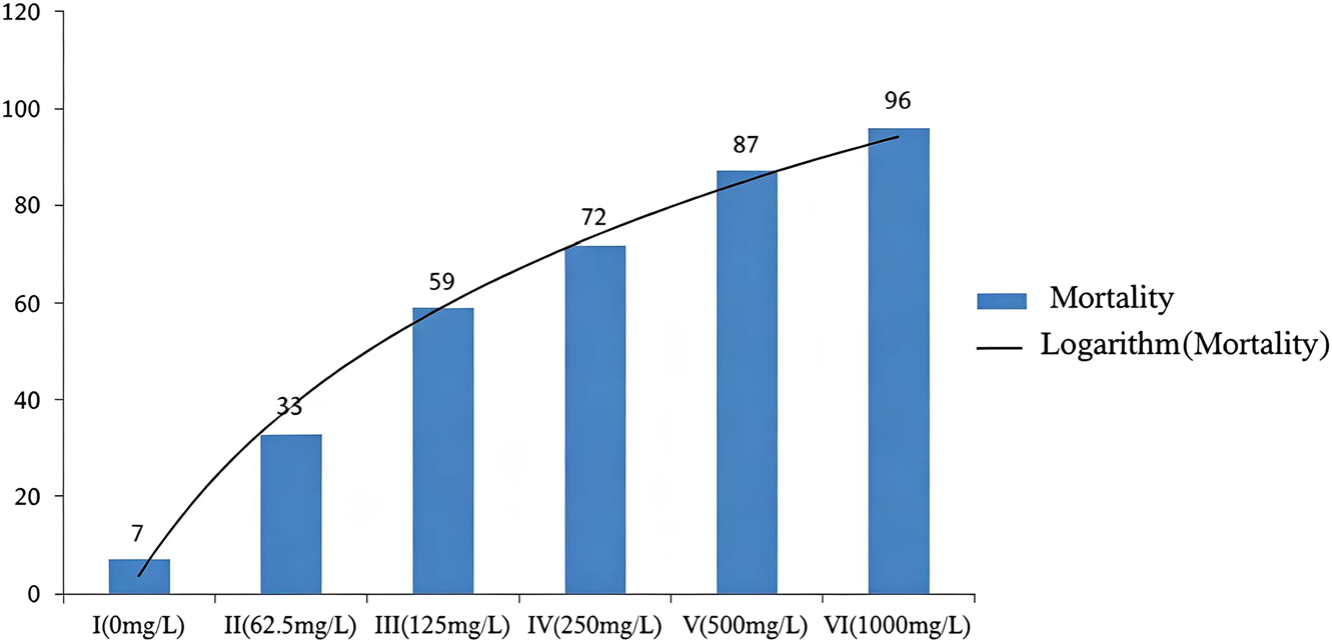
The mortality number of *R. microplus* in different concentrations of Deltamethrin solution.

The results (showed out Figure 1) of adult immersion test illustrates the mortality rates of *R. microplus* ticks exposed to varying concentrations of Deltamethrin solution. As shown in Figure 1, there is a clear variation in tick mortality across different Deltamethrin concentrations. In Group I, which had the lowest concentration, only 7 ticks died, likely due to natural causes. From Group II onwards, tick mortality increased markedly. Specifically, in Group II, 33 ticks died and 67 survived, yielding a mortality rate of 33.0 %. In Group III, the mortality rate rose to 59.0 %, with 59 ticks dying and 41 surviving. Group IV saw a further increase, with 72 ticks dying and 28 surviving, leading to a mortality rate of 72.0 %. In Group V, the mortality rate reached 87.0 %, as 87 ticks died and only 13 survived. Finally, in Group VI, the highest concentration tested, 96 ticks died and just 4 survived, resulting in a mortality rate of 96.0 %. Analyzing the trend line in Figure 1, it is evident that the most significant increase in tick mortality occurred between Groups I and II (from 0 to 62.5 mg/L). The next substantial increase was observed between Groups II and III (from 62.5 to 125 mg/L). Beyond this point, the rate of increase in tick mortality began to slow down, starting from Group III.

**Figure 2:**
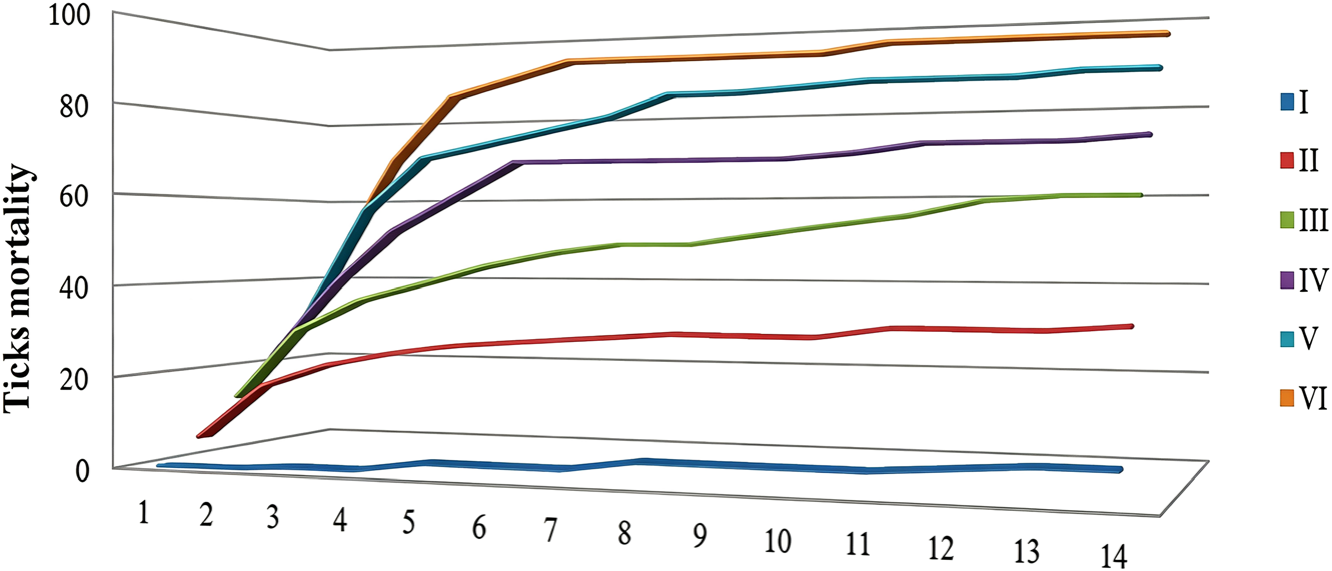
The dose-time rule of the number of death of *R. microplus* in different concentrations of Deltamethrin solution.

Additionally, the study examined the mortality patterns of *R. microplus* ticks exposed to varying concentrations of Deltamethrin solution, with the findings illustrated in Figure 2. From this figure, it is evident that a considerable proportion of ticks in each experimental group perished during the initial phase of the experiment. Following the ninth day, the rate of tick mortality plateaued, indicating no significant increase in the number of deaths thereafter.

### 3.2 Detection and analysis of Deltamethrin resistance in *R. microplus*

The lethal concentration (LC50) of Deltamethrin for *R. microplus* was determined using Graph Pad Prism 4 software, based on the mortality rates observed at various concentrations of Deltamethrin. The LC50 value was found to be 121.39 mg/L, with a 95 % confidence interval ranging from 99.08 to 148.73 mg/L. For the LC95, the lethal concentration was 952.61 mg/L, with a 95 % confidence interval between 944.02 and 1,148.15 mg/L. The regression analysis yielded an equation of *y* = 1.84*X* + 1.17, demonstrating a strong fit (*R* ^2^ = 0.96), a mortality slope of 0.64, and a resistance factor (RF) of 9.24. These findings suggest that *R. microplus* populations in Groups III to VI have developed significant resistance to Deltamethrin.

### 3.3 Amplification results of the sodium ion channel domain III gene in *R. microplus*

**Figure 3:**
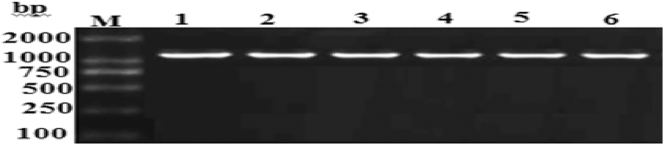
PCR amplification results of sodium channel domain III gene sequences of *R. microplus*.

The study results (showed out Figure 3) illustrates the PCR amplification results for the sodium ion channel domain III gene in six samples of *R. microplus*. As depicted, all randomly selected PCR products exhibited clear and distinct bands without any noticeable impurities. The amplified fragment size was approximately 1,100 bp, aligning with the expected target size.

### 3.4 Sequencing results, BLAST alignment, and ORF reading frame determination

By performing bidirectional sequencing and assembly, we obtained the initial sequence of the domain III sodium ion channel gene from *R. microplus*, which spans 1,067 base pairs. Individual BLAST alignments and analyses revealed that this sequence shares a similarity of 98–100 % with the published *R. microplus* sodium ion channel gene sequences in GenBank. Following translation, the open reading frame (ORF) of the domain III sodium ion channel gene was identified, measuring 1,010 base pairs in length.

### 3.5 Genetic evolutionary analysis

**Figure 4:**
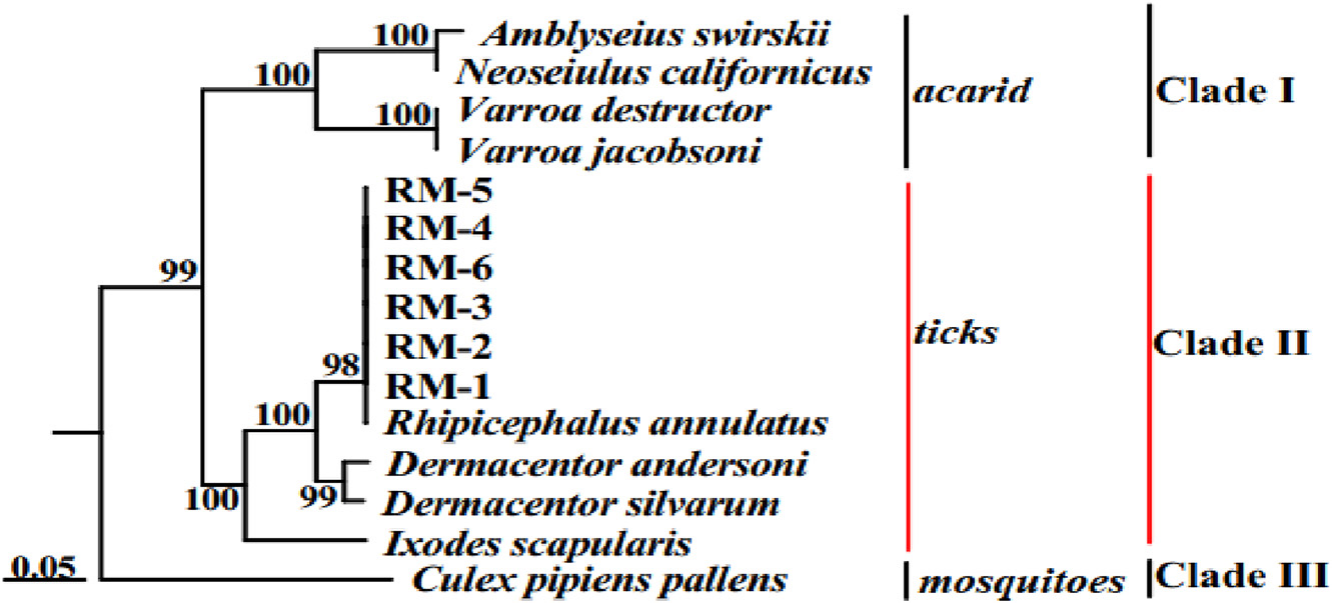
Genetic evolution tree of various genotypes of *R*. *microplus* by sodium channel domain III gene sequence.

Based on the ORF reading frame sequence of the domain III sodium ion channel gene, the genetic evolutionary relationship analysis results of *R. microplus* showed on Figure 4.

As illustrated in Figure 4, the genetic evolution tree is categorized into three primary clades (Clade I–III). Clade I includes *V. jacobsoni*, *V. destructor*, *N. californicus*, and *A. swirskii.* Notably, *V. jacobsoni* and *V. destructor* formed one subgroup within Clade I, supported by a nodal value of 100, whereas *N. californicus* and *A. swirskii* constituted another subgroup within Clade I, also with a nodal value of 100. Clade II comprises six strains of *R. microplus*, *R. annulatus*, *D. andersoni*, *D. silvarum*, and *I. scapularis*, which were obtained from this study. Specifically, *R. microplus* and *R. annulatus* formed the first subgroup of Clade II, with a nodal support of 98; *D. andersoni* and *D. silvarum* formed the second subgroup, supported by a nodal value of 99; and *I. scapularis* formed its own distinct branch within Clade II. Clade III is solely represented by *C. pipiens pallens*, forming an independent branch in this evolutionary tree.

### 3.6 Analysis of signal peptides, transmembrane domains, phosphorylation, and glycosylation sites in domain III protein in sodium ion channels

**Figure 5:**
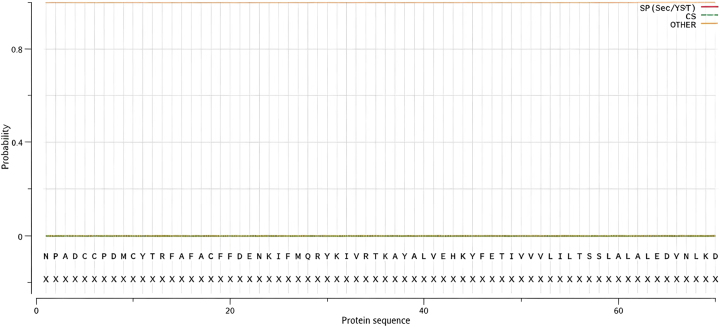
The signal peptide prediction results of sodium channel domain III protein of *R*. *microplus*.

The structural characteristics of the domain III protein within the sodium ion channel, including its signal peptide, transmembrane domains, phosphorylation sites, and glycosylation sites, were examined. Figure 5 illustrates the prediction results for the signal peptide of the domain III protein in the sodium ion channel of *R. microplus* ticks. As depicted in Figure 5, neither the drug-resistant (surviving ticks from groups Ⅲ–Ⅵ) nor the non-drug-resistant (surviving ticks from groups Ⅰ–Ⅱ) variants of the domain III protein in these ticks contain a signal peptide. This analysis provides insights into the structural composition of the domain III protein, which may contribute to a better understanding of its function and potential role in drug resistance mechanisms.

**Figure 6:**
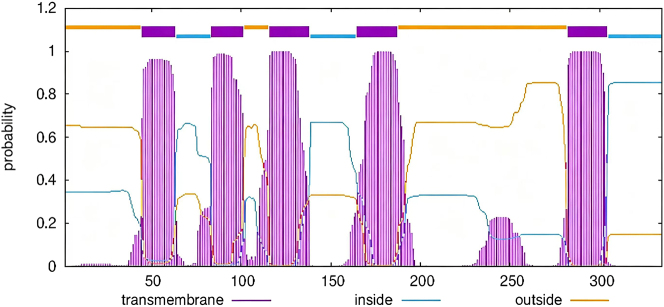
The transmembrane domain prediction results of sodium channel domain III protein of *R*. *microplus*.

Figure 6 illustrates the predicted transmembrane domains of the domain III protein of the sodium ion channel in *R. microplus* ticks. As shown in Figure 6, both the drug-resistant (surviving ticks from groups Ⅲ–Ⅵ) and non-drug-resistant (surviving ticks from groups Ⅰ–Ⅱ) variants of the domain III protein in these ticks exhibit six transmembrane domains. To further elaborate, the analysis reveals that regardless of whether the sodium ion channel protein displays drug resistance, it consistently contains six transmembrane regions. This structural characteristic is evident in both types of proteins examined.

**Figure 7:**
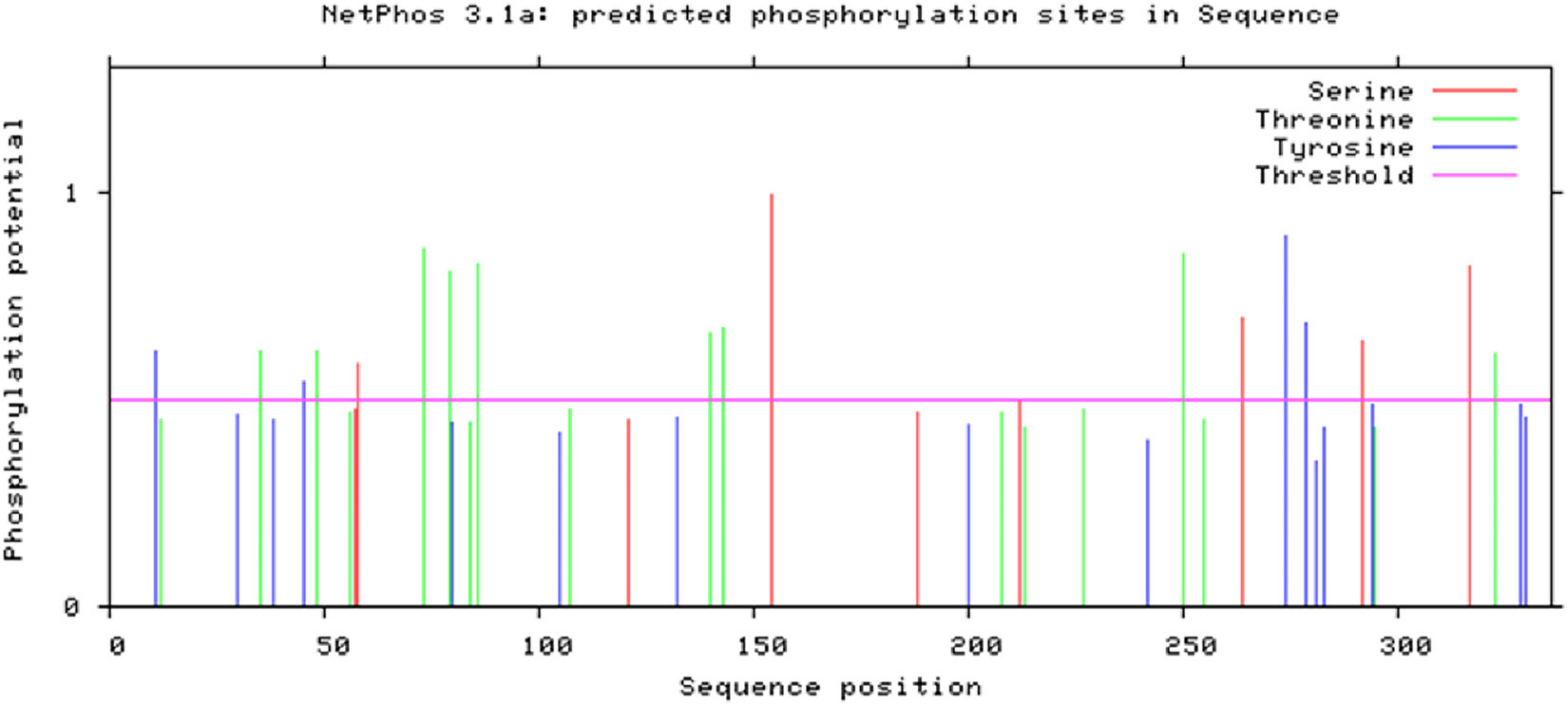
The phosphorylation sites prediction results of sodium channel protein of *R*. *microplus*.

The predicted phosphorylation sites in the domain III protein of the sodium ion channel of *R. microplus* ticks were shown in Figure 7. As illustrated in Figure 7, both the domain III proteins of the sodium ion channel of *R. microplus* ticks, whether resistant or susceptible to drugs, contained a total of 42 phosphorylation sites, including 9 serine phosphorylation sites, 18 threonine phosphorylation sites, and 15 tyrosine phosphorylation sites. In addition, Glycosylation sites prediction of domain III protein in sodium ion channels of *R. microplus* ticks was carried out. The results showed that the glycosylation sites within the domain III protein of the sodium ion channel in *R. microplus* ticks were identified. Analysis indicated that both resistant and non-resistant variants of this protein contain eight glycosylation sites, located at amino acid positions 9, 27, 97, 110, 112, 184, 228, and 254.

### 3.7 Tertiary structure analysis of domain III protein in sodium ion channels

**Figure 8:**
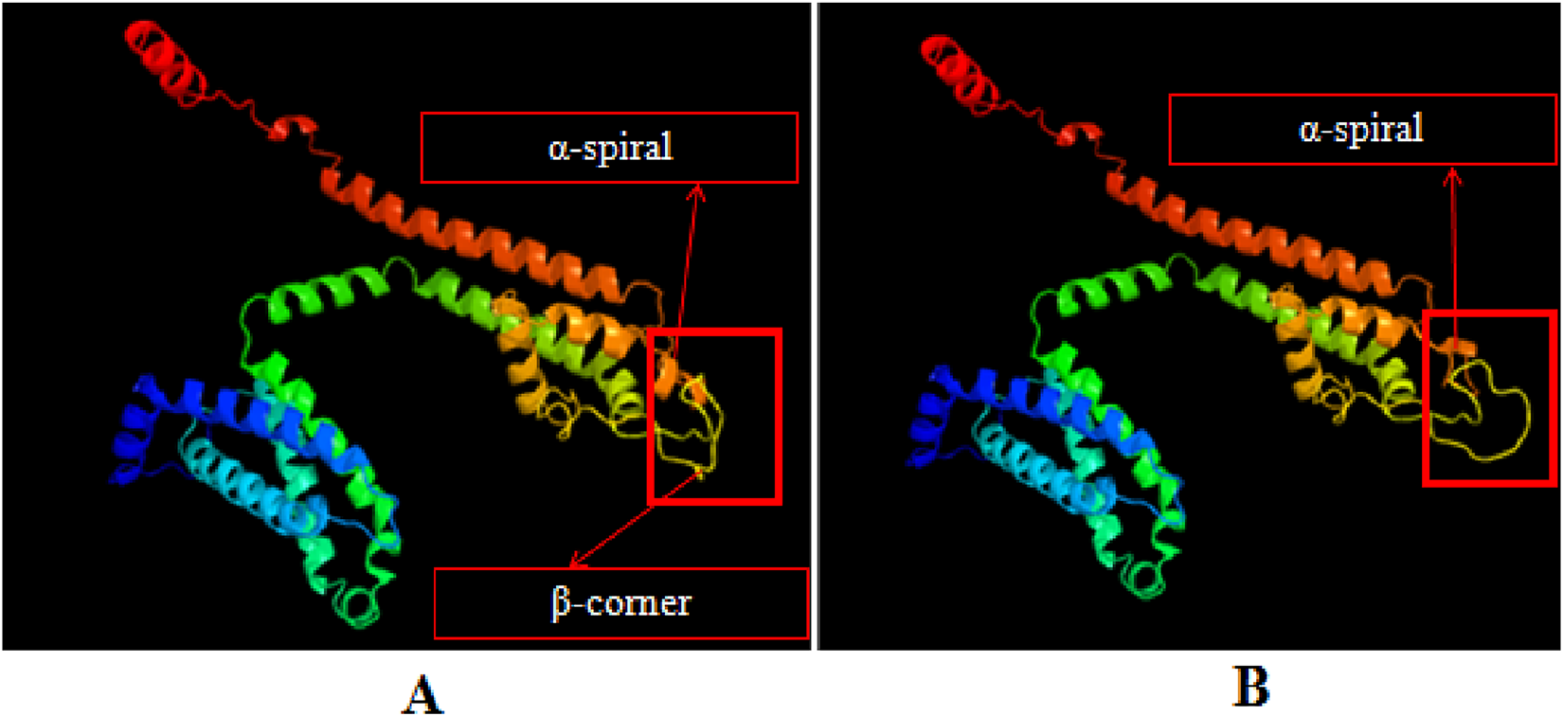
The sodium channel protein’ tertiary structure of *R*. *microplus*. Note: (A) The tertiary structure of domain III protein in the sodium channel of *R*. *microplus* without showing resistance to Deltamethrin. (B) The tertiary structure of the domain III protein in the sodium channel of *R*. *microplus* showing resistance to Deltamethrin.

Predictions were made regarding the tertiary structures of the domain III protein in the sodium ion channels of both drug-resistant and non-resistant *R. microplus* ticks. The findings are illustrated in Figure 8. As shown, the tertiary structures of both variants consist of three main elements: α-helices, β-turns, and random coils. Notably, α-helices dominate, with both N- and C-termini adopting this conformation. Comparisons between the non-resistant domain III protein and models c5gjvA, c7vfvA, and c8f6pA revealed coverage rates of 92.0 %, 90.0 %, and 87.0 %, respectively, with a consistent similarity of 30.0 %. For the resistant variant, when compared to models c3jbrA, c7vfvA, and c5gjvA, the coverage rates were 92.0 %, 92.0 %, and 90.0 %, respectively, with similarities of 31.0 %, 30.0 %, and 29.0 %. Figure 8 highlights notable differences in the tertiary structure between the two variants (marked by red boxes). Specifically, the random coil region in the non-resistant variant is more compact and includes a β-turn. Conversely, the random coil in the resistant variant is more extended and lacks a β-turn. Additionally, the α-helix in the non-resistant variant is significantly longer in this region compared to the resistant variant.

### 3.8 Detection of base nutation sites and analysis of their association with resistance generation results

Sequence alignment revealed that two consecutive base mutations (at the 938th and 939th nucleotide positions) were identified in the ORF reading frame of the sodium ion channel gene in Deltamethrin-resistant *R. microplus* ticks. No such mutations were observed in non-resistant ticks from groups Ⅰ–Ⅱ. By integrating tick from groups Ⅲ–Ⅵ resistance data and the tertiary structure analysis of the sodium ion channel domain III protein, it was determined that these mutations led to the substitution of phenylalanine with isoleucine, altering the tertiary structure of domain III and possibly contributing to tick resistance. Consequently, this study concluded that the detected base mutations are directly linked to Deltamethrin resistance in *R. microplus*.

## 4 Discussion

In recent years, as tick sensitivity to pesticides has declined, resistance has become a significant global research focus [25]. *R. microplus*, an ectoparasitic arthropod prevalent in tropical and subtropical regions [26], has exhibited varying degrees of resistance across multiple countries and regions [27], including Brazil, India, Australia, South Africa, and Somalia [28], [29], [30], [31], [32]. Two primary methods for detecting tick resistance are LPT and AIT [33]. While the FAO-endorsed LPT method is considered the standard, its lengthy process (5–6 weeks) has limited its adoption in some areas [34], 35]. In contrast, the AIT method, which takes approximately one week, has been widely used for resistance testing. Literature indicates that mutations in the sodium ion channel gene correlate with parasite resistance phenotypes. For instance, a mutation at position 1,014 in *C. pipiens pallens* (A to T, leucine to phenylalanine) confers Deltamethrin resistance [36]. Similarly, Cruz-Valdés et al. found that G184C, C190A, and T2134A mutations in *R. microplus* influence cypermethrin resistance [35].

The adult immersion test results showed that mortality rates in *R. microplus* increased with higher Deltamethrin concentrations. However, the rate of increase slowed after groups II–III, eventually stabilizing. Notably, some ticks survived even at higher concentrations, particularly in groups V and VI, suggesting Deltamethrin resistance. This may indicate that these ticks originated from cattle farms where Deltamethrin is frequently used as a livestock insecticide. Furthermore, the relationship between mortality and time in different Deltamethrin concentrations revealed that while mortality did not increase proportionally with time, there was a gradual rise over extended periods. This suggests that Deltamethrin resistance in *R. microplus* is a result of long-term evolutionary adaptation rather than rapid development. Combining these findings, it appears that prolonged and frequent use of Deltamethrin in Huaihua area cattle farms has contributed to tick resistance. Although LC50, LC95, and their confidence limits do not provide detailed information on resistant or sensitive individuals within populations, they confirm the presence and extent of resistance [37]. Some researchers advocate for further exploration of these values to better understand regional tick resistance [38]. In this study, LC50 and LC95 values confirmed Deltamethrin resistance in Huaihua-area *R. microplus*. Based on the RF value (9.24, within 5.1–25.0), and following established criteria, *R. microplus* in Huaihua exhibited II-level resistance, indicating moderate Deltamethrin resistance due to frequent pesticide use.

Genetic evolution analysis revealed that the ORF reading frame sequence of the sodium ion channel gene varies among arthropods. As shown in Figure 4, *R. microplus* clustered with *R. annulatus*, *D. andersoni*, *D. silvarum*, and *I. scapularis* in Clade II, with *R. microplus* and *R. annulatus* forming a closely related sub-branch. This suggests high homology between these species’ sodium ion channel genes.

Analysis of the signal peptide, transmembrane domains, phosphorylation, and glycosylation sites of domain III of the sodium ion channel protein indicated that *R. microplus* lacks a signal peptide but possesses transmembrane domains, phosphorylation, and glycosylation sites. This implies that the protein is involved in intracellular ion transport rather than signal transduction. With 42 phosphorylation sites, the protein undergoes phosphorylation during synthesis, regulating intracellular metabolism and enzyme activity. Glycosylation aids in rapid polypeptide chain folding in the endoplasmic reticulum, ensuring correct protein structure and function.

Tertiary structure predictions and base mutation analyses revealed significant differences between drug-resistant and non-resistant *R. microplus* ticks. These differences are primarily due to base mutations in the ORF reading frame, leading to amino acid substitutions and structural changes in the protein. Literature supports that such structural changes often confer knockdown resistance (KDR) [30]. In this study, sequencing and comparison of the domain III gene in resistant *R. microplus* revealed two adjacent base mutations causing phenylalanine-to-isoleucine conversion, consistent with findings by He and Nogueira [4], 21].Therefore, the author believes that the base mutation sites detected in the domain III gene sequence of the sodium ion channel of *R. microplus* are directly related to the resistance of the mutant genotype of *R. microplus* to Deltamethrin.

## 5 Conclusions

This study presents the first investigation of Deltamethrin resistance in *R. microplus* ticks collected from the Huaihua region of Hunan Province. Results from this study demonstrated a level II resistance to Deltamethrin in the local tick population. Moreover, sequence analysis of the voltage-gated sodium channel gene domain III in resistant ticks revealed two base mutation sites, which are possibly contributing to the development of resistance.
